# Antioxidant and Hepatoprotective Activities of Polysaccharides from Spent Mushroom Substrates (*Laetiporus sulphureus*) in Acute Alcohol-Induced Mice

**DOI:** 10.1155/2017/5863523

**Published:** 2017-12-21

**Authors:** Huajie Zhao, Yufei Lan, Hui Liu, Yongfa Zhu, Weiru Liu, Jianjun Zhang, Le Jia

**Affiliations:** ^1^College of Life Science, Shandong Agricultural University, 271018 Tai'an, China; ^2^Tai'an Academy of Agricultural Sciences, 271000 Tai'an, China; ^3^The Second High School of Tai'an, 271000 Tai'an, China

## Abstract

In order to contribute to the exploitation and utilization of spent mushroom substrates (SMS) of *Laetiporus sulphureus*, hot-water-extractable polysaccharides (H-SMPS) and enzymatic-extractable polysaccharides (E-SMPS) were successfully isolated from SMS of *L. sulphureus*. Both H-SMPS and E-SMPS were found to have high reducing power and potential scavenging activities against hydroxyl, DPPH, and superoxide anion radicals. *In vivo* assays showed that the administration of H-SMPS and E-SMPS has potential hepatoprotective effects against alcohol-induced alcoholic liver disease (ALD), possibly brought about by improving liver function, increasing antioxidant status, and reducing lipid peroxidation. Furthermore, monosaccharide composition analysis showed that fucose may play a vital role in guaranteeing stronger hepatoprotection. These results may provide references for the exploitation of the SMS of *L. sulphureus* as a source of H-SMPS and E-SMPS, which in turn can be used as functional foods or natural drugs for the prevention of ALD and other liver diseases.

## 1. Introduction

With the prevalence of poor diet structures in China, increasing alcohol consumption has been demonstrated to be involved in the increasing incidence of alcoholic liver disease (ALD), which is a progressive disease that could subsequently induce hepatitis, hepatic fibrosis, cirrhosis, and liver cancer [1–3]. Furthermore, ALD could also cause other clinical diseases including hypertension, hyperlipidemia, and atherosclerosis [4, 5]. Alcohol metabolism mainly occurs in the liver, and many studies have indicated that many *in vivo* enzymes, such as alcohol dehydrogenase (ADH), aldehyde dehydrogenase (ALDH), and cytochrome P4502E1 (CYP2E1), are involved in alcohol metabolism [6, 7]. The toxicity of acute alcohol consumption in the liver is possibly due to the inhibition of ADH and ALDH activities, as well as the increase in CYP2E1 activity, which is accompanied by the excessive formation of reactive oxygen species (ROS) [5, 8]. Increasing amounts of evidence indicate that ROS could oxidize biological membranes, proteins, nucleic acids, and other biological macromolecules, thereby damaging cellular integrity and functionality [9]. At present, the mechanisms underlying acute alcohol metabolism and its metabolic consequences are still poorly understood. The traditional therapies for ALD are mainly abstinence and the administration of nutritional supplements and corticosteroid substances; however, their therapeutic effects have been proven to be poor [10]. Recently, natural substances from edible and medicinal mushrooms have attracted attention as potential drugs in preventing and treating alcohol-induced liver injury [11]. Hence, it seems necessary and significant to explore natural and nontoxic therapeutic strategies for the treatment of ALD.

It has been shown that more than five million tons of spent mushroom substrates (SMS), the cultivation matrix that remains after mushroom cultivations, are produced in China annually with low utilization, causing environmental pollution and resource waste [12]. SMS contains residual mycelia and fruiting bodies of mushrooms; trace elements including Fe, Ca, Zn, and Mg; active enzymes such as cellulose, hemicellulose, and ligninase; and biomacromolecules including polysaccharides, proteins, and lipids [12, 13]. Polysaccharides extracted from SMS have received attention owing to their abundant pharmacological activities including antioxidation, hepatoprotection, and antihyperlipidemia [14, 15]. Additionally, these polysaccharides possess less toxic side effects and are more accessible locally and more environment friendly, compared to synthetic substances [14].

*Laetiporus sulphureus*, an edible and medicinal fungus belonging to *Basidiomycetes*, is widely distributed in Asia, Europe, and North America [16]. Documents in literature have indicated that intracellular polysaccharides and exopolysaccharides from *L. sulphureus* show antioxidant, anti-inflammatory, and antitumor activities [17–19]. However, studies on the hepatoprotective activities of polysaccharides isolated from *L. sulphureus* SMS are scarce. In this work, the hepatoprotective effects and antioxidant activities of hot-water-extractable SMPS (H-SMPS) and enzymatic-extractable SMPS (E-SMPS) from *L. sulphureus* SMS in acute alcohol-induced ALD mice were investigated.

## 2. Materials and Methods

### 2.1. Materials and Chemicals

SMS of *L. sulphureus* was obtained from Tai'an Agricultural Sciences Academy (Tai'an, China). Kunming strain mice (male) were purchased from Taibang Biologic Products Co. Ltd., Tai'an, China. Monosaccharide standard samples were provided by Sigma Chemicals Co. Ltd., St. Louis, USA The diagnostic kits for analyzing the activities of ADH, ALDH, CYP2E1, superoxide dismutase (SOD), glutathione peroxidase (GSH-Px), and catalase (CAT), as well as the levels of malondialdehyde (MDA), lipid peroxidation (LPO), total cholesterol (TC), and triglyceride (TG), were purchased from Jiangsu Meibiao Biological Technology Co. Ltd., Jiangsu, China. All other reagents used in this experiment were provided by local chemical suppliers.

### 2.2. Preparation of H-SMPS and E-SMPS

The SMS of *L. sulphureus* was dried at 55°C and ground into powder. H-SMPS and E-SMPS were extracted by incubating the SMS in deionized water (90°C, 8 h) and snailase solutions (4% *w*/*v*, 37°C, 6 h), respectively. After centrifugation (3000 rpm, 10 min), the supernatant was collected, concentrated, and precipitated by incubation with three volumes of 95% *v*/*v* ethanol at 4°C overnight. The precipitate was deproteinated by the Sevage method [20] and dialyzed against deionized water.

### 2.3. Animal Experiments

Animal procedures were performed in accordance with the institutional ethical guidelines for animals' welfare of Shandong Agricultural University Committee. Seventy male Kunming strain mice (20 ± 2 g) were housed in an animal room under standardized environmental conditions (temperature of 23 ± 2°C, a relative humidity of 50 ± 5%, and a 12/12 h light/dark cycle) for 5 days with free access to food and water.

After domestication, the mice were randomly divided into seven groups of ten mice each. These groups were the normal control (NC) group, model control (MC) group, positive control (PC) group (received bifendatatum at 150 mg/kg), H-SMPS high-dose group (400 mg/kg), H-SMPS low-dose group (100 mg/kg), E-SMPS high-dose group (400 mg/kg), and E-SMPS low-dose group (100 mg/kg). The mice in the NC and MC groups received isometric normal saline until the 25th day as a blank. On the 26th day, all mice except those in the NC group received alcohol (50% *v*/*v*) at a dose of 12 mL/kg by gavages. All mice were sacrificed quickly by euthanasia overnight after inducing toxicity with alcohol. The animal experiment design thought was in accordance with previous literature [6, 21, 22].

Blood samples were collected and centrifuged (10,000 rpm, 10 min, 4°C) to obtain the sera. Alanine aminotransferase (ALT) activity and aspartate aminotransferase (AST) activity in the sera were measured using an automatic biochemical analyzer (BS-380, Shenzhen, China).

The liver was surgically removed, weighed, and homogenized (10% *w*/*v*) in phosphate buffer solution (0.2 M, pH 7.4). After centrifugation (3000 rpm, 10 min, 4°C), the supernatant was collected for further biochemical assay. The hepatic activities of ADH, ALDH, CYP2E1, SOD, GSH-Px, and CAT, as well as the levels of MDA, LPO, TC, and TG, were determined using commercial reagent kits following the manufacturer's instructions. The liver samples used for histopathological observations were soaked in formalin (10% *w*/*v*). Thin sections (4-5 *μ*m thickness) were acquired using a microtome and stained with hematoxylin and eosin. Hepatic histopathological changes were observed and photographed using a microscope under ×400 magnification.

### 2.4. *In Vitro* Antioxidant Analysis

The reducing powers of H-SMPS and E-SMPS were determined using the methods reported by Oyaizu [23]. The reaction system, which contained 1 mL of either of the polysaccharides (0–800 *μ*g/mL), 2.5 mL of phosphate buffer (0.2 M, pH 6.6), and 1.0 mL of potassium ferricyanide (1% *w*/*v*), was kept warm in a water bath at 50°C for 20 min. The reaction was terminated by adding 2 mL of trichloroacetic acid (10% *w*/*v*) and 1.2 mL of ferric chloride (0.1% *w*/*v*). The absorbance was measured at 700 nm using deionized water as a blank control and butylated hydroxytoluene (BHT) as a positive control.

The scavenging activities of H-SMPS and E-SMPS against hydroxyl radicals were measured according to a reported method [24]. The reaction mixture, composed of 1 mL of ferrous sulfate (9 mM), 1 mL of salicylic acid (9 mM), 1 mL of either of the polysaccharides (0–800 *μ*g/mL), and 1 mL of hydrogen peroxide (8.8 mM), was incubated for 30 min at 37°C. After centrifugation (12,000 rpm, 6 min), the absorbance was measured at 510 nm using deionized water as a blank control and BHT as a positive control. The scavenging rate was calculated using the following formula:

(1) $$\begin{array}{c} Scavenging\;rate\;\left(\%\right)=\left[\frac{A_{0}-A_{1}}{A_{0}}\right]\times 100, \end{array}$$

where *A*_0_ is the absorbance of the blank and *A*_1_ is the absorbance of the polysaccharides or BHT.

The DPPH radical scavenging activities of H-SMPS and E-SMPS were measured using the method reported by Cheng et al. [25]. The reaction mixture, which contained either of the polysaccharides (0.2 mL, 0–800 *μ*g/mL) and DPPH solution (0.6 mL, 0.004% *w*/*v* in methanol), was stored in the dark for 30 min without moving. The absorbance was measured at 517 nm using deionized water as a blank control and BHT as a positive control. The scavenging rate was evaluated as follows:

(2) $$\begin{array}{c} Scavenging\;rate\;\left(\%\right)=\left[\frac{A_{0}-A_{1}}{A_{1}}\right]\times 100, \end{array}$$

where *A*_0_ is the absorbance of the blank control and *A*_1_ is the absorbance of the polysaccharides or BHT.

Superoxide anion radical scavenging activities were determined using the method reported by Wei et al. [26]. The reaction system, composed of 1 mL of either of the polysaccharides (0–800 *μ*g/mL) and 2 mL of Tris-HCl buffer (50 mM, pH 8.2), was incubated for 20 min at 25°C. After incubation, 0.4 mL of 1,2,3-phentriol (5 mM) was added to terminate the reaction. The absorbance was measured at 325 nm using deionized water as a blank control and BHT as a positive control. The scavenging rate was calculated using the following equation:

(3) $$\begin{array}{c} Scavenging\;rate\;\left(\%\right)=\left[\frac{A_{0}-A_{1}}{A_{0}}\right]\times 100, \end{array}$$

where *A*_0_ is the absorbance of the blank and *A*_1_ is the absorbance of polysaccharides or BHT.

The IC_50_ values (*μ*g/mL) were defined as the effective concentrations of the sample at which the radicals were inhibited by 50%.

### 2.5. Monosaccharide Composition Determination

Monosaccharide compositions of H-SMPS and E-SMPS were analyzed by gas chromatography (GC-2010, Shimadzu, Japan) equipped with a flame ionization detector (FID) according to a reported method [27]. Briefly, 0.1 g of the sample was hydrolyzed with 1.8 mL of trifluoroacetic acid (2 M) at 100°C for 4 h. The resulting hydrolysate was neutralized with 0.6 mL of ammonium hydroxide (12 M), restored with 0.4 mL of sodium borohydride in ammonium hydroxide (2% *w*/*v*), and acetylated with 0.6 mL of methylimidazole and 4 mL of acetic anhydride. After centrifugation (3000 rpm, 10 min), 1 *μ*L of the supernatant was injected into a capillary column of Rtx-1 (30 mm × 0.25 mm × 0.25 *μ*m). Monosaccharide components were evaluated based on the standard curves of rhamnose, arabinose, xylose, mannose, galactose, glucose, ribose, and fucose.

### 2.6. Statistical Analysis

The software SAS was used to perform statistical evaluation. Data are expressed as mean ± SD (standard deviations). One-way ANOVA was performed to analyze the data. Significant differences between experimental groups were determined using Tukey's tests. *P* < 0.05 was considered as the threshold for statistical significance.

## 3. Results

### 3.1. Effects of H-SMPS and E-SMPS on AST and ALT Activities in Sera

**Figure 1:**
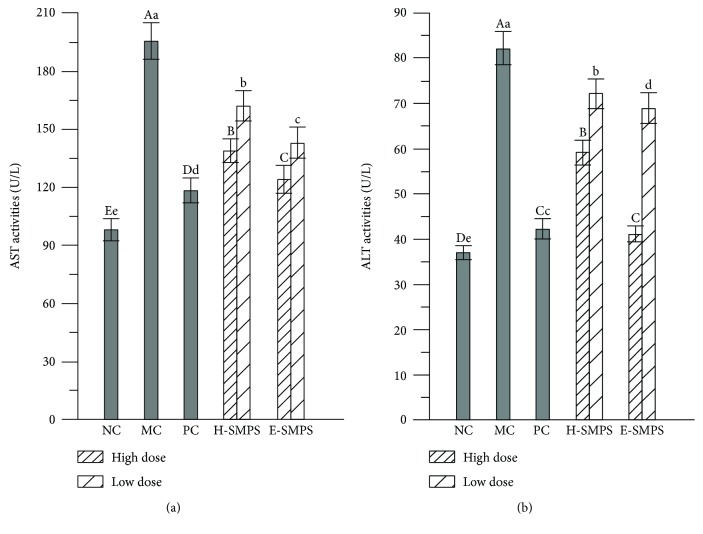
Effects of H-SMPS and E-SMPS on the serum activities of AST (a) and ALT (b). The values were reported as means ± SD. Bars with different letters were significantly different (*P* < 0.05).

The mice in the MC group showed abnormally higher serum AST and ALT activities than the mice in the NC group (*P* < 0.05, Figure 1), indicating that alcohol-induced liver injury was successfully established in mice. Interestingly, the elevation of AST and ALT activities could be attenuated by supplementation of H-SMPS and E-SMPS, especially at high doses. Compared to mice in the MC group, the serum AST and ALT activities of mice in the H-SMPS high-dose group decreased by 28.29% and 28.0%, respectively, while those of mice in the E-SMPS high-dose group decreased by 36.6% and 50.0% of mice, respectively. In addition, bifendatatum was also observed to decrease the serum enzyme activities. These results demonstrate that both H-SMPS and E-SMPS have the potential to suppress acute alcohol-induced elevation of AST and ALT activities in order to maintain liver function.

### 3.2. Effects of H-SMPS and E-SMPS on Hepatic Lipid Properties

**Figure 2:**
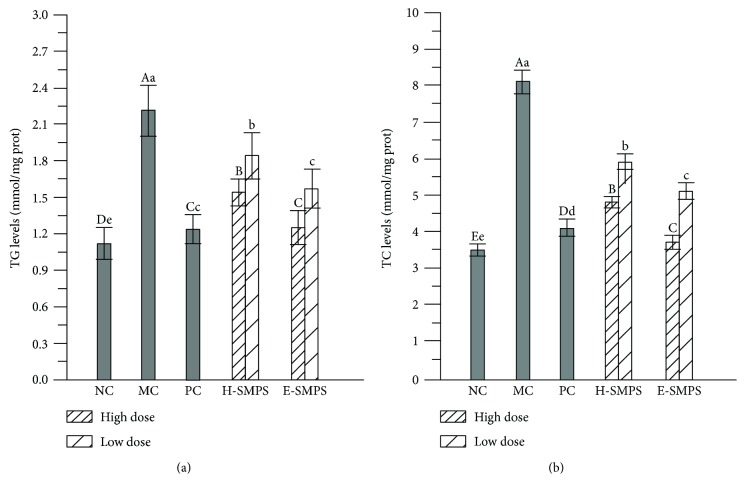
Effects of H-SMPS and E-SMPS on hepatic lipid profiles of TG (a) and TC (b). The values were reported as means ± SD. Bars with different letters were significantly different (*P* < 0.05).

As shown in Figure 2, the mice in the MC group exhibited higher hepatic TG and TC levels than the mice in the NC group (*P* < 0.05), indicating that hepatic lipid metabolic disturbance was induced by acute alcohol toxicity. The administration of H-SMPS and E-SMPS attenuated the elevation of TG and TC levels, especially with E-SMPS administered at a high dose. Briefly, the hepatic TG and TC levels in mice treated with a high dose of E-SMPS reached 1.25 ± 0.14 and 3.7 ± 0.18 mmol/mg prot, respectively, which were lower than those observed in the H-SMPS group at the same dose (TG: 1.54 ± 0.11 mmol/mg prot and TC: 4.8 ± 0.16 mmol/mg prot), and were almost equal to those in the NC group (TG: 1.12 ± 0.13 mmol/mg prot and TC: 3.5 ± 0.16 mmol/mg prot). The present results indicate that both H-SMPS and E-SMPS could potentially restore lipid metabolism to its state before acute alcohol-induced toxicity.

### 3.3. Effects of H-SMPS and E-SMPS on Hepatic ADH and ALDH Activities and CYP2E1 Level

**Figure 3:**
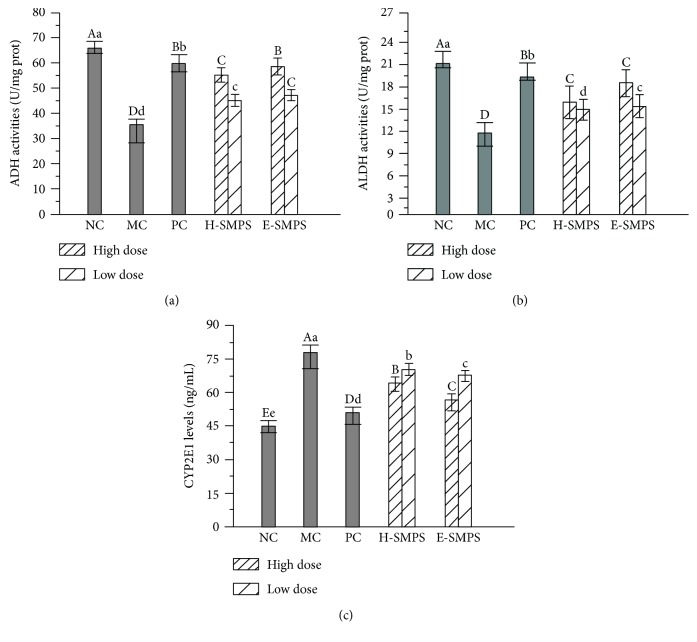
Effects of H-SMPS and E-SMPS on alcohol-metabolizing enzyme activities of ADH (a) and ALDH (b) as well as levels of CYP2E1 (c). The values were reported as means ± SD. Bars with different letters were significantly different (*P* < 0.05).

The hepatic ADH and ALDH activities and CYP2E1 levels are shown in Figure 3. Compared with mice in the NC group, the mice in the MC group had decreased ADH and ALDH activities and increased CYP2E1 levels (*P* < 0.05), indicating that alcohol metabolism was partly suppressed in the alcohol dehydrogenase oxidation system and accelerated in the microsome alcohol oxidation system in response to acute alcohol-induced toxicity. Interestingly, the remarkable elevation in ADH and ALDH activities and reduction in CYP2E1 levels could be observed when H-SMPS and E-SMPS are administered at the tested dosages (*P* < 0.05). The ADH and ALDH activities of mice treated with a high dose of E-SMPS reached maximum values of 58.9 ± 3.4 and 18.5 ± 1.9 U/mg prot, respectively, which were higher than those of mice in the H-SMPS group (ADH: 55.3 ± 2.8 U/mg prot and ALDH: 15.9 ± 2.1 U/mg prot) at the same dose. Additionally, compared with the CYP2E1 levels in the H-SMPS high-dose group (64.30 ± 2.72 ng/mL), the CYP2E1 levels in the E-SMPS high-dose group were lower (56.73 ± 2.98 ng/mL).

Meanwhile, bifendatatum-treated mice also exhibited an increase in the activities of ADH and ALDH, as well as an attenuation in the CYP2E1 levels when compared with the MC group mice (*P* < 0.05).

### 3.4. Effects of H-SMPS and E-SMPS on Hepatic Antioxidative Enzymes

**Figure 4:**
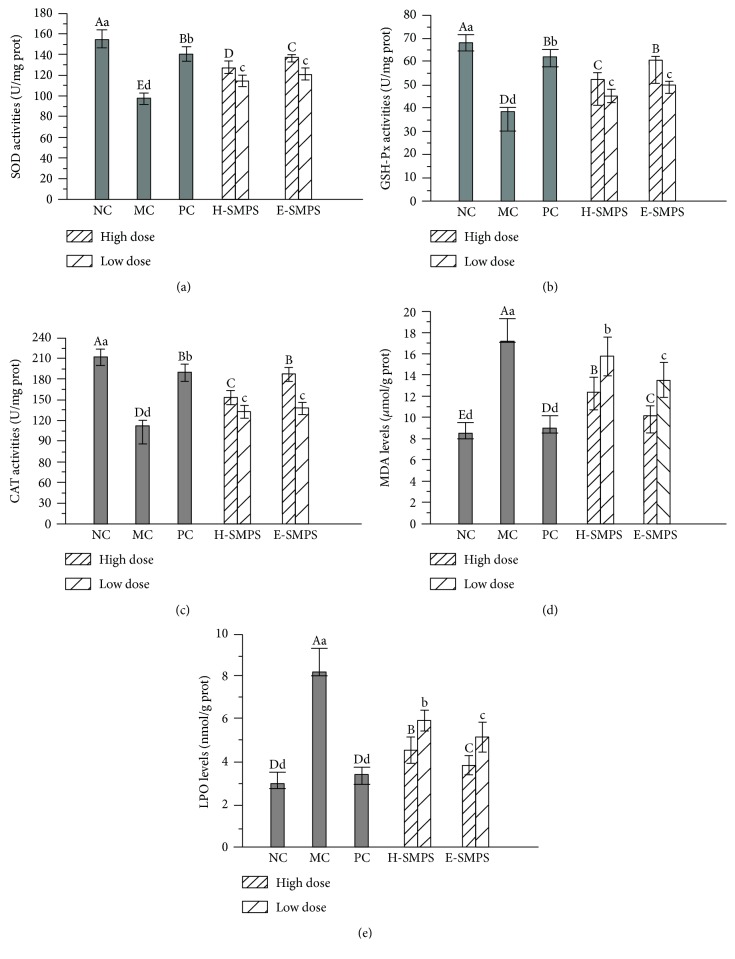
Effects of H-SMPS and E-SMPS on antioxidant enzymes of SOD (a), GSH-Px (b), and CAT (c), as well as lipid peroxides of MDA (d) and LPO (e). The values were reported as means ± SD. Bars with different letters were significantly different (*P* < 0.05).

Compared with the mice in the NC group, the mice in the MC group were observed to have an abnormal reduction in hepatic SOD, GSH-Px, and CAT activities (*P* < 0.05, Figures 4(a)–4(c)), indicating that the hepatic antioxidative defense had been damaged by the administration of alcohol. The changes in these parameters were ameliorated by treatment with H-SMPS, E-SMPS, and bifendatatum at all doses (with all *P* < 0.05). The hepatic SOD, GSH-Px, and CAT activities in the H-SMPS group were elevated by 30.6%, 36.8%, and 44.6%, respectively, compared with those in the MC group. In the E-SMPS high-dose group, hepatic SOD, GSH-Px, and CAT activities were elevated by 40.8%, 58.4%, and 54.5%, respectively, compared with those in the MC group. Based on the present results, H-SMPS, E-SMPS, and bifendatatum can potentially restore the damaged antioxidative defense.

### 3.5. Effects of H-SMPS and E-SMPS on Hepatic Lipid Peroxidation

In the MC group, the hepatic lipid peroxidation of MDA and LPO was remarkably increased by 104.8% and 159.4%, respectively, as compared to the NC group (*P* < 0.05, Figures 4(d)–4(e)). This indicates that oxidative stress had occurred in the liver as a result of the administration of alcohol. However, treatment with H-SMPS and E-SMPS significantly mitigated the abnormal increases in MDA and LPO levels (*P* < 0.05). A high dose of H-SMPS inhibited MDA and LPO levels by 27.9% and 41.0%, respectively, while E-SMPS inhibited MDA and LPO levels by 40.7% and 50.6%, respectively, when compared with levels in the MC group. These results demonstrate that both E-SMPS and H-SMPS could protect the liver against oxidative stress by inhibiting lipid peroxidation. Furthermore, the MDA and LPO levels in the PC group were almost equal to those in the NC group.

### 3.6. Effects of H-SMPS and E-SMPS on Hepatic Histological Changes

**Figure 5:**
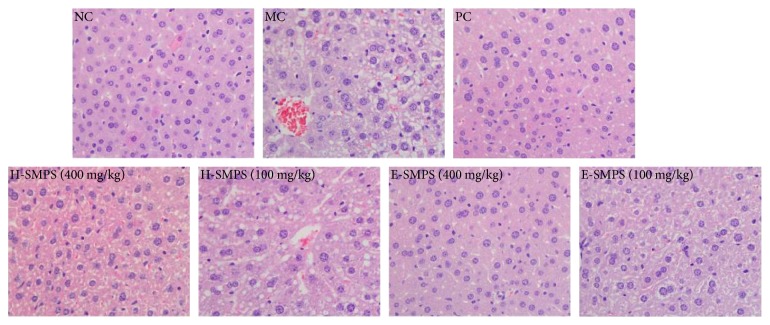
Effects of H-SMPS and E-SMPS on liver damages in acute alcohol-induced mice.

Images of hepatic histological sections are displayed in Figure 5. No obvious histological changes were observed in the livers of mice in the NC group. In contrast, hepatic sections of mice in the MC group showed nucleus contraction, loss of cellular boundaries in the cytoplasm of hepatocytes, necrotic hepatocytes around the central vein, and massive fatty tissues. Interestingly, the hepatic sections of the mice treated with H-SMPS and E-SMPS showed obvious improvement compared to the mice in the MC group. In particular, the mice treated with a high dose of E-SMPS had hepatic sections similar to those from the NC group, indicating that H-SMPS and E-SMPS could protect liver tissue from acute alcohol-induced hepatic injury.

### 3.7. Antioxidant Activities *In Vitro*

**Figure 6:**
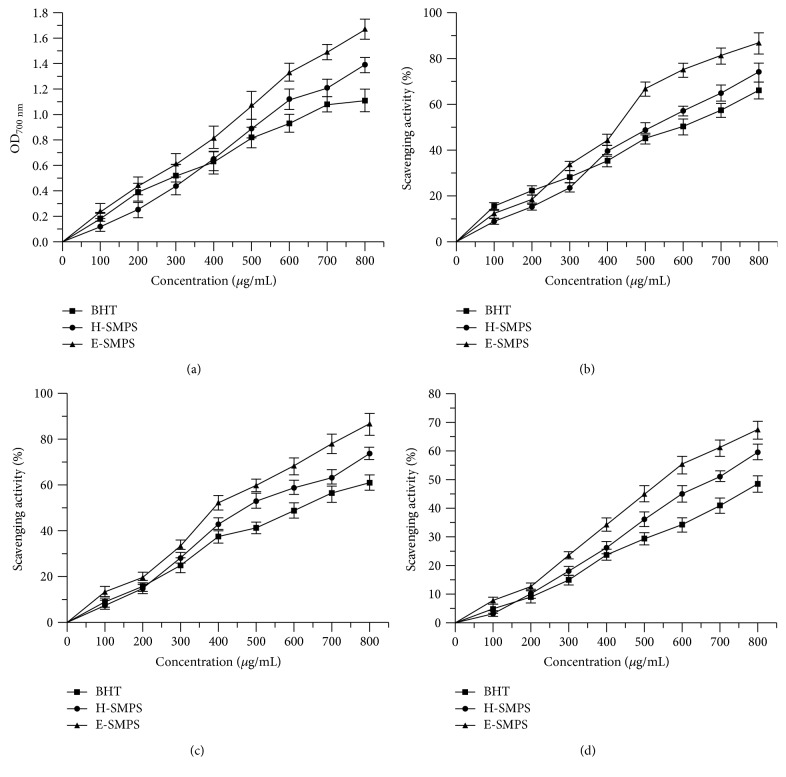
*In vitro* antioxidant activities of H-SMPS and E-SMPS. Reducing power (a) and scavenging activities on hydroxyl radicals (b), DPPH radicals (c), and superoxide anion radicals (d).

The reducing power of H-SMPS and E-SMPS increased as the polysaccharide concentration increased from 0 to 800 *μ*g/mL (Figure 6(a)). At a concentration of 800 *μ*g/mL, the reducing power of E-SMPS (1.67 ± 0.08) was higher than those of H-SMPS (1.39 ± 0.06) and BHT (1.11 ± 0.09).

The scavenging activities of E-SMPS and H-SMPS against hydroxyl radicals were shown to be dose dependent (Figure 6(b)). At a concentration of 800 *μ*g/mL, the hydroxyl radical scavenging activities of H-SMPS, E-SMPS, and BHT reached 74.10 ± 4.11%, 86.6 ± 4.52%, and 66.22 ± 3.77%, respectively. Moreover, the IC_50_ values of H-SMPS, E-SMPS, and BHT were found to be 500.31 ± 2.70, 375.75 ± 2.58, and 564.64 ± 2.75 *μ*g/mL, respectively, indicating that E-SMPS has stronger scavenging activities against hydroxyl radicals than H-SMPS does.

The color of DPPH solutions was reduced with the enhancement of antioxidant activities. H-SMPS and E-SMPS showed observable scavenging activities against DPPH radicals following an increase in the concentrations of the polysaccharides (Figure 6(c)). At a concentration of 800 *μ*g/mL, the DPPH scavenging activity of E-SMPS reached 86.6 ± 4.73%, which was higher than those of H-SMPS (73.88 ± 4.25%) and BHT (61.27 ± 3.41%). The IC_50_ values of H-SMPS (479.90 ± 2.68 *μ*g/mL), E-SMPS (378.12 ± 2.58 *μ*g/mL), and BHT (604.12 ± 2.78 *μ*g/mL) were consistent with the above results.

The superoxide radical scavenging activities of H-SMPS, E-SMPS, and BHT are shown in Figure 6(d). A positive correlation between scavenging activity and concentration is seen. The scavenging activities of H-SMPS, E-SMPS, and BHT against superoxide radicals were 59.51 ± 2.84%, 67.22 ± 3.11%, and 48.40 ± 2.91%, respectively. Furthermore, the IC_50_ values of H-SMPS, E-SMPS, and BHT were 674.35 ± 2.83, 553.09 ± 2.72, and 868.62 ± 2.94 *μ*g/mL, respectively, indicating that E-SMPS displays stronger superoxide radical scavenging activity than H-SMPS and BHT.

### 3.8. Monosaccharide Composition Analysis

**Figure 7:**
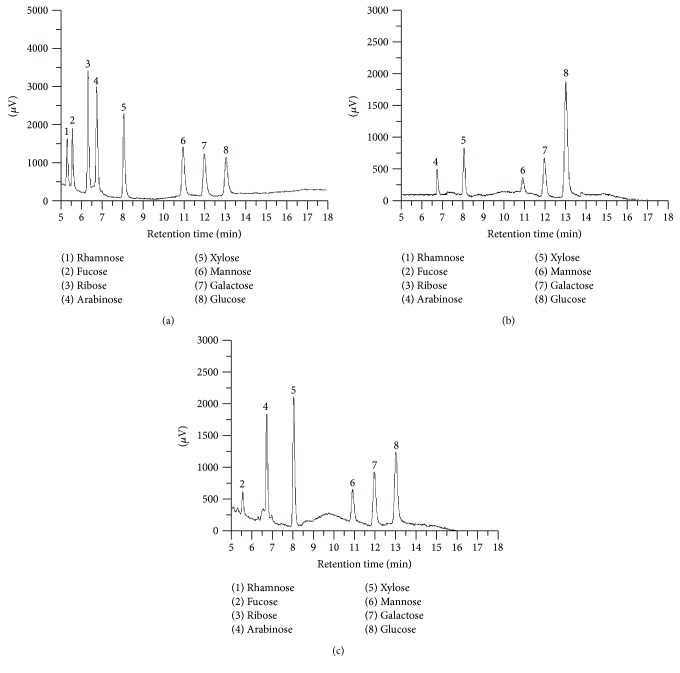
GC chromatograms of standard monosaccharides (a), H-SMPS (b), and E-SMPS (c).

The monosaccharide compositions of H-SMPS and E-SMPS were determined by comparing their retention times with those of reference monosaccharides (Figure 7(a)). As seen in Figures 7(b) and 7(c), H-SMPS is composed of arabinose, xylose, mannose, galactose, and glucose, with mass percentages of 4.45%, 10.08%, 6.78%, 17.22%, and 61.37% (Figure 7(b)), respectively. However, E-SMPS was found to be composed of fucose, arabinose, xylose, mannose, galactose, and glucose, with mass percentages of 6.88%, 15.04%, 22.71%, 8.81%, 18.43%, and 28.13% (Figure 7(c)), respectively.

## 4. Discussion

In recent years, the use of polysaccharides from fungus as natural medicines has gained increasing attention owing to the lack of negative consequences in using them as treatments against many pathological diseases, as compared to chemical synthetic drugs. Several publications have established many polysaccharide extraction methods including hot water, alkaline, acidic, enzymatic, ultrasonic, and microwave extractions [28–32]. Increasing amounts of evidence have shown that enzymatic-extractable polysaccharides from edible mushrooms possess high antioxidant and biological activities. The use of these polysaccharides has many other advantages such as high extraction yields, reproducibility of results, low energy consumption, and low pollution [30, 33]. However, few reports on the exploration of the antioxidant and hepatoprotective activities of E-SMPS in alcohol-induced mice have been published.

As an organ that is sensitive to cytotoxicity during chemotherapy, the liver plays important roles in alcohol metabolism [6]. Acute alcohol consumption could induce the formation of poisonous metabolic products, causing abnormalities in alcohol metabolism, thereby leading to liver damage [34]. Clinically, serum enzymes (AST and ALT), hepatic lipids (TC and TG), and enzymes involved in alcohol metabolism (ADH, ALDH, and CYP2E1) are commonly used as biochemical markers for early diagnosis of hepatic injury. Serum ALT and AST activities are elevated when hepatic injury occurs since ALT and AST could leach out of hepatocytes into the blood circulation. This is associated with ballooning degeneration, massive centrilobular necrosis, and cellular infiltration [35, 36]. Furthermore, acute alcohol-induced lipid metabolism disorders, including accumulation of fat and lipochondrion on the surface of hepatocytes, could be reflected in abnormal changes of hepatic TC and TG levels. *In vivo*, alcohol metabolism mainly consists of two pathways, the alcohol dehydrogenase oxidation system and the microsome alcohol oxidation system, catalyzed by the metabolic enzymes ADH, ALDH, and CYP2E1 [6]. During alcohol consumption, about 90% of the alcohol can be metabolized into acetaldehyde by ADH, which is further metabolized into acetic acid by ALDH, and eventually decomposed into carbon dioxide and water. About 8–10% of alcohol is metabolized by CYP2E1 in the tricarboxylic acid cycle [37, 38]. The activities of ADH and ALDH, two crucial enzymes in regulating alcohol metabolism, could be remarkably suppressed when acute alcohol consumption occurs [5]. The activity of CYP2E1, an important metabolic enzyme in catalyzing exogenous and endogenous compounds that plays a role in hepatic alcohol metabolism, could be increased when alcohol accumulation activates the microsome alcohol oxidation system [34]. In the present study, the ALD mice in the MC group showed serious liver damage as evidenced by significant increases in serum enzyme activities (AST and ALT), hepatic CYP2E1 levels, and hepatic lipid levels (TC and TG), as well as decreases in hepatic ADH and ALDH activities, when compared with the mice in the NC group. The abnormal changes were remedied by the supplementation of H-SMPS and E-SMPS, indicating that polysaccharides from the SMS of *L. sulphureus* have potential protective effects against toxicity induced by acute alcohol consumption. Furthermore, the hepatoprotective effects of H-SMPS and E-SMPS were confirmed by observation of histopathological sections.

It has been reported that acute alcohol consumption can induce oxidative stress *in vivo*, leading to the overproduction of ROS. This plays an important role in the development of ALD due to the toxic effects of ROS on lipids, enzymes, nucleic acids, and proteins [39–41]. Hence, it seems necessary to increase the scavenging abilities against ROS. Previous literature has indicated that reducing power, which reflects the electron donation capacity of a compound, is one of the most important indicators for evaluating antioxidant activity [42]. The hydroxyl radicals, which are the major type of radicals, have been proven to be involved in the toxicity against biomolecules and in the induction of lipid peroxidation. Hence, there is great potential in discovering natural antioxidant agents with higher hydroxyl radical scavenging activities for the development of treatments against radical-induced diseases [43]. Scavenging activities against DPPH radicals, which are stable free radicals, are a wide indicator and allow a rapid method for assessing antioxidant activities [44]. Superoxide anion radicals, which are relatively weak oxidants, are one of the precursors of singlet oxygen and hydroxyl radicals and, as such, could indirectly activate lipid peroxidation and amplify cellular damage [45]. Based on the present results, both H-SMPS and E-SMPS showed significant reducing power and scavenging activities against hydroxyl, DPPH, and superoxide anion radicals. Previous studies have proposed that antioxidant enzymes, including SOD, GSH-Px, and CAT, are the primary defense in eliminating ROS-induced oxidative stress *in vivo* [46]. The possible underlying mechanism may be the degradation of superoxides into H_2_O_2_ by SOD and the subsequent decomposition to O_2_ and H_2_O by CAT and GSH-Px, which results in the suppression of ROS generation [47]. Moreover, accumulated studies have proven that ALD is also connected with lipid peroxidation, which is also regarded as an indicator of oxidative damage [48, 49]. In our *in vivo* assays, significant decreases in SOD, GSH-Px, and CAT activities as well as remarkable increases in MDA and LPO levels were observed after alcohol consumption, indicating that liver function had been damaged and the antioxidant defense mechanism had been disabled [50]. The polysaccharide supplements significantly remedied the decreased enzyme activities and increased lipid peroxidation, demonstrating that both H-SMPS and E-SMPS have the potential to alleviate acute alcohol-induced liver damage.

The monosaccharide compositions of polysaccharides from *Catathelasma ventricosum* [46], *Lentinus edodes* [12], *T. albuminosus* [27], and *Flammulina velutipes* [14] were found to be different from the monosaccharide compositions of H-SMPS and E-SMPS from the SMS of *L. sulphureus* determined in the present study. This difference may be attributed to the differences in strains, culture methods, and extraction conditions used. Furthermore, previous studies have demonstrated that the biological functions of polysaccharides were mainly determined by their monosaccharide compositions [51]. Only fucose could be observed in E-SMPS, indicating that fucose may play an important role in conferring higher biological activities. Additionally, Schneider et al. also revealed that fucose plays a vital role in maintaining biological functions such as immunoregulation and anticancer activities in mammals [52].

## 5. Conclusions

The *in vitro* and *in vivo* analyses in this study demonstrate that both H-SMPS and E-SMPS confer effective hepatoprotection against acute alcohol-induced ALD, possibly by reducing oxidative stress. E-SMPS showed superior effects, indicating that enzymatic hydrolysis has a potential effect on enhancing bioactivities. The results may provide a mechanistic basis for the use of polysaccharides from *L. sulphureus* as a potential natural and functional food supplement for the prevention and alleviation of ALD and its complications.

## Abbreviations

ADH: Alcohol dehydrogenase
ALT: Alanine aminotransferase
ALD: Alcoholic liver disease
ALDH: Aldehyde dehydrogenase
AST: Aspartate aminotransferase
BHT: Butylated hydroxytoluene
CAT: Catalase
CYP2E1: Cytochrome P4502E1
E-SMPS: Enzymatic-extractable polysaccharides
FID: Flame ionization detector
GSH-Px: Glutathione peroxidase
H-SMPS: Hot-water-extractable polysaccharides
LPO: Lipid peroxidation
MC: Model control
MDA: Malondialdehyde
NC: Normal control
PC: Positive control
ROS: Reactive oxygen species
SD: Standard deviations
SMS: Spent mushroom substrates
SOD: Superoxide dismutase
TC: Total cholesterol
TG: Triglyceride.
